# Autonomic imbalance and atrial ectopic activity—a pathophysiological and clinical view

**DOI:** 10.3389/fphys.2022.1058427

**Published:** 2022-12-02

**Authors:** Alina Scridon

**Affiliations:** Physiology Department, Center for Advanced Medical and Pharmaceutical Research, University of Medicine, Pharmacy, Science and Technology “George Emil Palade” of Târgu Mureș, Târgu Mureș, Romania

**Keywords:** atrial arrhythmias, atrial ectopy, atrial fibrillation, autonomic nervous system, neuromodulation

## Abstract

The heart is one of the most richly innervated organs and the impact of the complex cardiac autonomic network on atrial electrophysiology and arrhythmogenesis, including on atrial ectopy, is widely recognized. The aim of this review is to discuss the main mechanisms involved in atrial ectopic activity. An overview of the anatomic and physiological aspects of the cardiac autonomic nervous system is provided as well as a discussion of the main pathophysiological pathways linking autonomic imbalance and atrial ectopic activity. The most relevant data on cardiac neuromodulation strategies are emphasized. Unanswered questions and hotspots for future research are also identified.

## Introduction

**FIGURE 1:**
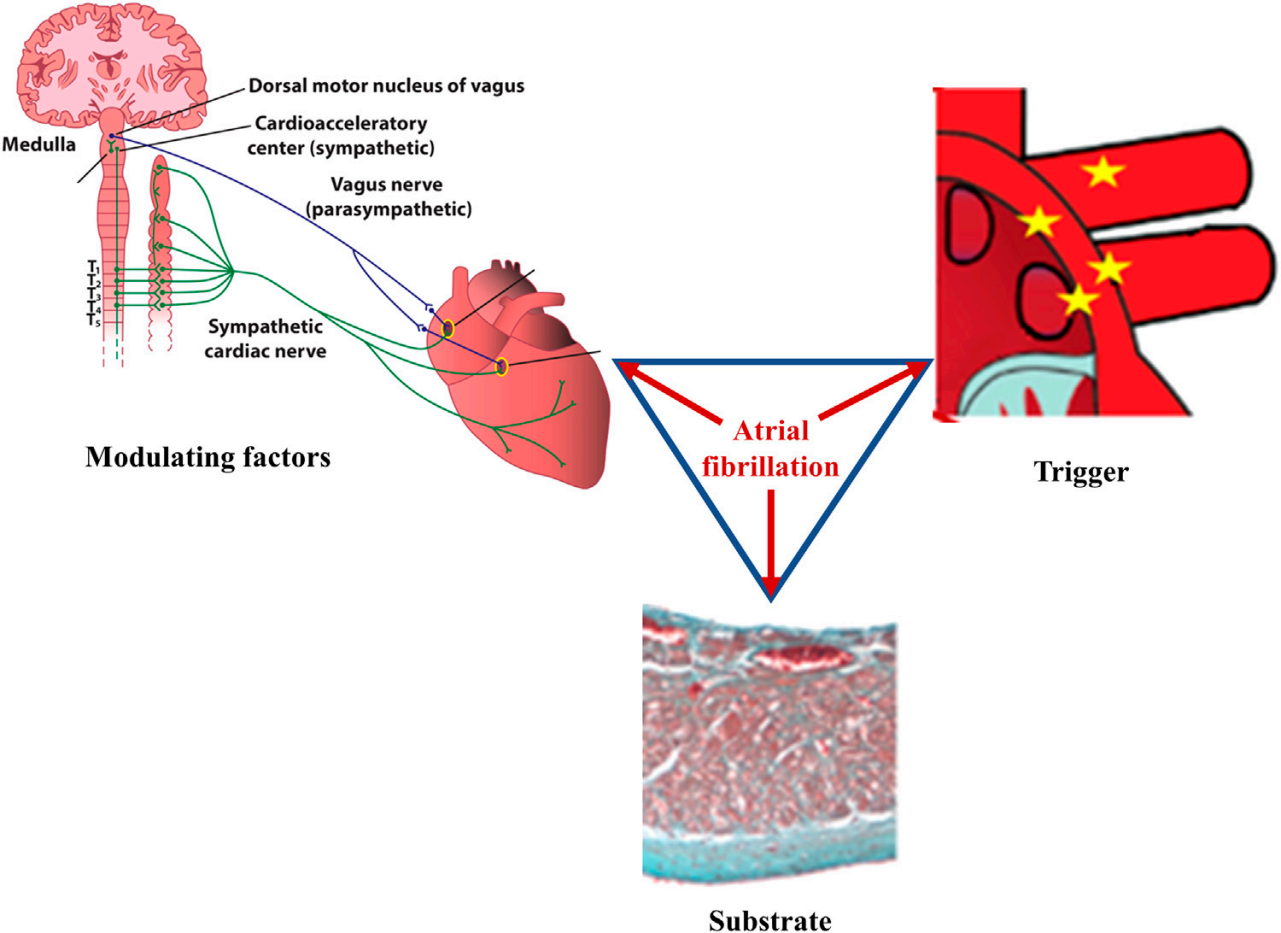
Main factors underlying atrial fibrillation. According to Coumel, three factors are needed for sustained cardiac arrhythmias to occur: abnormal triggers, substrate, and modulating factors. In atrial fibrillation, ectopic foci, most commonly located at the junction between the left atrium and the pulmonary veins, generate rapid, chaotic, and non-coordinated electrical impulses that initiate multiple reentry circuits; this component represents the arrhythmic trigger. If present, arrhythmogenic electrical and/or structural changes of the atria will act as the substrate that ensures persistence of the arrhythmia. Autonomic imbalance is currently recognized as the main pro-arrhythmogenic modulating factor, favoring both the initiation and the maintenance of the arrhythmia. Once it begins, atrial fibrillation favors its own perpetuation (“atrial fibrillation begets atrial fibrillation”) by inducing pro-arrhythmic electrical, structural, and autonomic remodeling of the atria.

**FIGURE 2:**
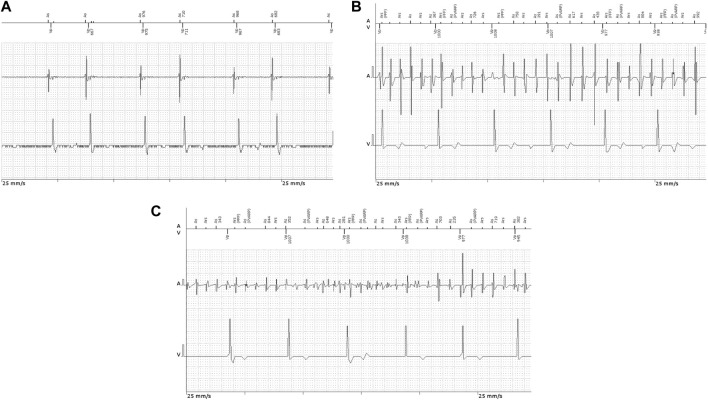
Intracavitary ECG recordings in patients implanted with permanent pacemakers showing **(A)** atrial couplets and **(B,C)** atrial high-rate episodes.

According to Coumel’s triangle concept, abnormal triggers, arrhythmogenic substrate, and modulating factors are needed to ensure the occurrence of sustained cardiac arrhythmias (Figure 1), including atrial fibrillation (AF) (Coumel, 1993). Autonomic imbalance is currently regarded as the most important pro-arrhythmic modulating factor, favoring both atrial ectopic activity and reentry (Scridon et al., 2018). In the presence of extensively remodeled atria, even infrequent ectopic activity can initiate sustained, persistent or permanent AF. However, in the absence of significant substrate that can support reentry, triggers sensitive to autonomic modulation will generate only unsustained atrial arrhythmias, including isolated or more complex premature atrial contractions (PACs), or short, self-terminating episodes of clinically overt or subclinical AF (Figure 2). (Scridon et al., 2018)

The aim of this review is to discuss the main mechanisms involved in atrial ectopic activity. An overview of the anatomic and physiological aspects of the cardiac autonomic nervous system (ANS) is provided as well as a discussion of the main pathophysiological pathways linking autonomic imbalance and atrial ectopic activity. The most relevant data on atrial neuromodulation strategies are emphasized.

## Ectopic automaticity and afterdepolarizations–the players behind the atrial ectopy scene

**FIGURE 3:**
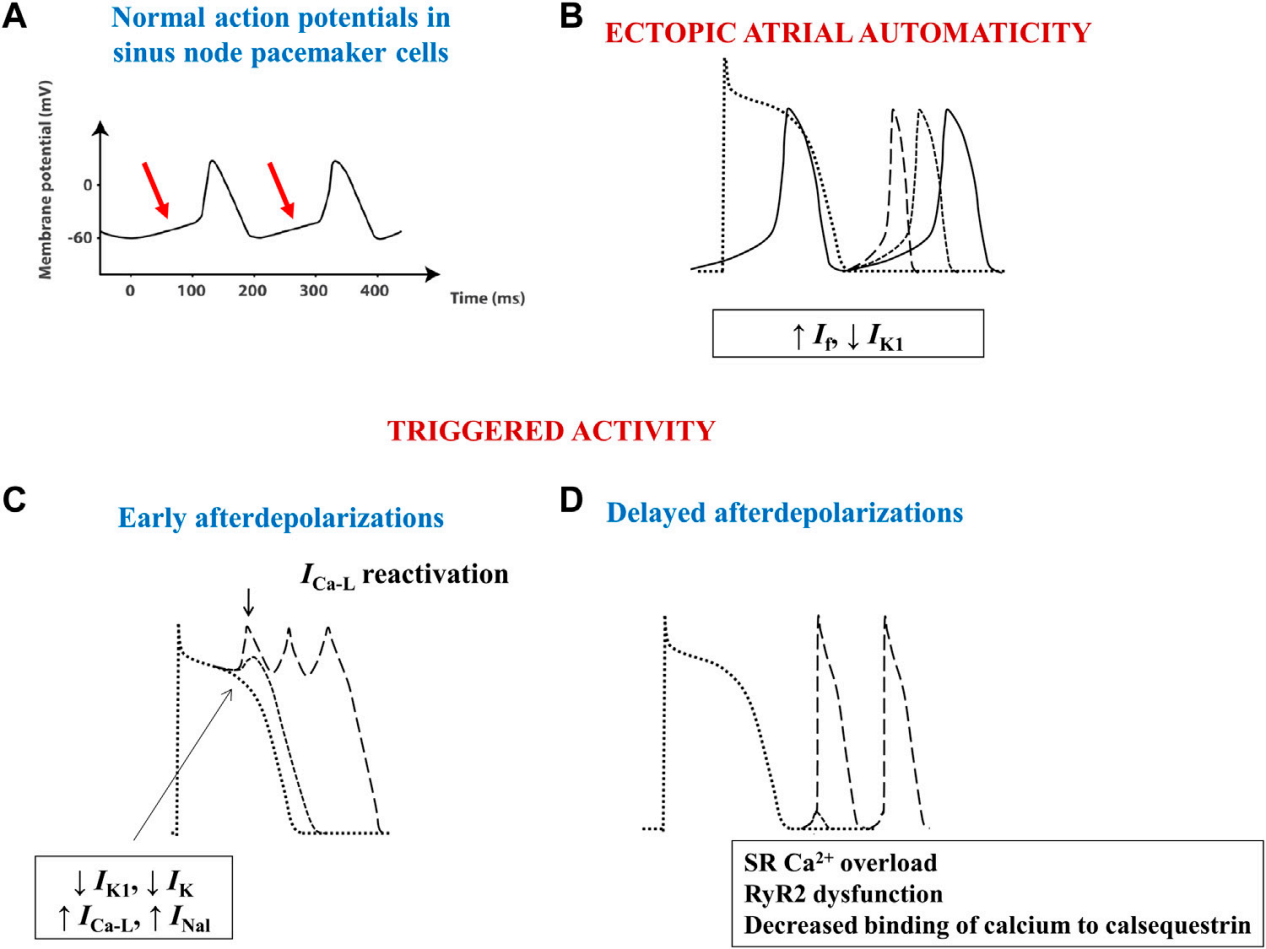
Schematic images of normal and arrhythmia-related atrial electrical activity. **(A)** Normal slow-response action potential in cardiac pacemaker cells. The arrows indicate the spontaneous diastolic depolarization phase. **(B)** Ectopic automaticity arises as a result of an increase in pacemaker current (*I*_f_) activity, a decrease in inwardly rectifying Kir current (*I*_K1_) activity, or a combination of both, capable of transforming atrial myocytes into pacemaker-like cells. **(C)** Excessive action potential prolongation due to a decrease in outward potassium currents (*I*_K1_ and *I*_K_) activity, an increase in inward (L-type calcium–*I*_Ca-L_–or late sodium - *I*_Nal_) current activity, or a combination of both, allows calcium channels to recover their ability to activate, creating inward cation movement during action potential phases 2 or 3, thus generating early afterdepolarizations and ectopic atrial activity. **(D)** Spontaneous release of calcium from the sarcoplasmic reticulum can occur during phase 4 of the action potential due to sarcoplasmic reticulum calcium overload, decreased binding of calcium to calsequestrin, or to increased cardiac ryanodine-receptor channel activity. The consequent intracellular calcium overload further activates the Na^+^/Ca^2+^ exchanger, creating a net inward cation movement, thus providing the basis for delayed afterdepolarizations and for ectopic atrial activity. RyR2—cardiac ryanodine-receptor channels; SR–sarcoplasmic reticulum.

Physiologically, the electrical activity of the heart arises as a result of spontaneous diastolic depolarization of sinoatrial node pacemaker cells (Figure 3A). Three main mechanisms have been shown to underlie the advent of abnormal triggers and of atrial ectopy: enhanced ectopic automaticity, early (phase 2 or 3) and delayed (phase 4) afterdepolarizations (Figure 3B–D). In AF, the trigger is often related to ectopic foci located at or in the close vicinity of the pulmonary venous ostia, although other areas, such as the right atrium or the left atrial posterior wall, the ostium of the superior vena cava or of the coronary sinus, crista terminalis, or the ligament of Marshall can also act as origin of abnormal atrial electrical activity (Wu et al., 2001).

Similarly to pacemaker cells, normal cardiac myocytes also express the pacemaker current–*I*_f_. These cells do not behave as pacemaker cells because the activity of *I*_f_ is much lower and because they concomitantly express the inwardly rectifying Kir current, *I*_K1_, that opposes *I*_f_, preventing the spontaneous depolarization of these cells. However, a decrease in the activity of *I*_K1_, an increase in the activity of *I*_f_, or both, can easily transform these cells into pacemaker cells that will generate abnormal, ectopic electrical impulses (Figure 3B). Indeed, in rats with spontaneous AF one of the most upregulated genes was *HCN4*, encoding for proteins of *I*_f_ (Scridon et al., 2014), whereas *in vitro* studies performed in canine AF models have shown a significant increase in *I*_f_ activity and spontaneous diastolic depolarization rate, which were both counteracted by the *I*_f_ blocker ivabradine (Li et al., 2015). In normal rats, chronic ivabradine administration induced a significant increase in atrial *HCN4* mRNA expression levels (Scridon et al., 2021), suggesting that *I*_f_ blockade may exert not anti-, but rather proarrhythmic effects over the long term, as also indicated by the modest, yet significant increase in AF rates recorded in the clinical studies that evaluated ivabradine (Martin et al., 2014; Cammarano et al., 2016). Human patients with sinus node dysfunction may also develop AF (tachy-brady syndrome).

Ectopic activity can also arise when cardiomyocytes develop spontaneous, progressive depolarizations, that interrupt phases 2, 3, or 4 of the action potential. Excessive action potential prolongation, most commonly due to decreased repolarizing potassium currents activity, allows calcium channels to recover their activatability, creating inward cation movement during action potential phases 2 or 3, thus generating early afterdepolarizations and ectopic atrial activity (Figure 3C). Although early afterdepolarizations can act as triggers in certain cases, delayed, phase 4 afterdepolarizations seem to be the most important source of atrial ectopic activity. Spontaneous release of calcium from the sarcoplasmic reticulum (SR) can occur through cardiac ryanodine-receptor channels (RyR2) during phase 4 of the action potential due to SR calcium overload, decreased binding of calcium to calsequestrin (the main calcium-binding protein of the SR), or to increased RyR2 activity (Scridon et al., 2018). The consequent intracellular calcium overload further activates the Na^+^/Ca^2+^ exchanger (NCX), which creates a net inward movement of positive charges, thus setting the basis for delayed afterdepolarizations and for ectopic atrial activity (Figure 3D).

## An anatomic and physiological view on the autonomic innervation of the atria

### Anatomy of the cardiac autonomic nervous system

**FIGURE 4:**
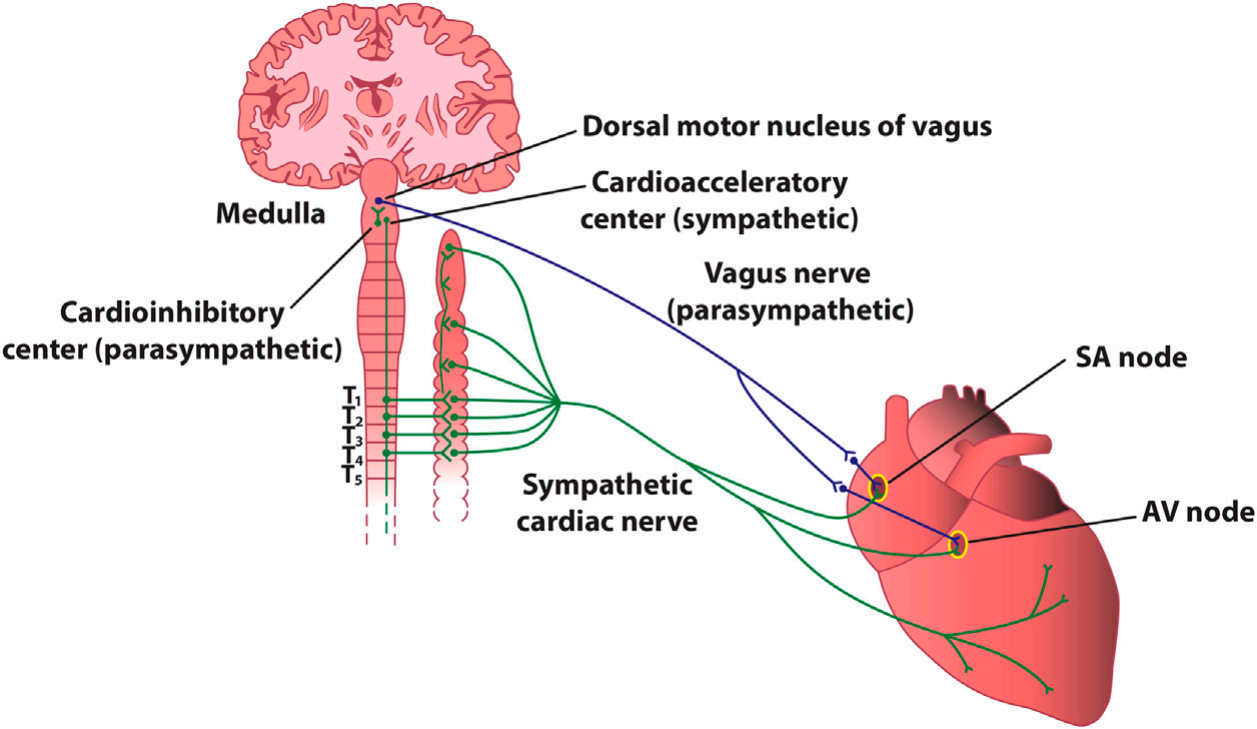
Stylized diagram of the anatomy of cardiac sympathetic and parasympathetic innervation. The figure presents schematically some of the most important connections between the autonomic nervous system and the heart. In reality, sympathetic and parasympathetic projections are less spatially distinct, and overlap at various levels. For instance, the parasympathetic innervation of the atrioventricular node and of the inferior atrium is primarily *via* fibers originating in the inferior vena cava-right atrium, and that of the ventricles is primarily *via* fibers originating in the ventral interventricular artery ganglionated plexi. AV–atrioventricular; SA–sinoatrial.

The heart contains very rich intrinsic and extrinsic autonomic innervation that provides physiological regulation of the heart rate and hemodynamic parameters, as well as cellular and subcellular properties of individual cardiac cells (Shen and Zipes, 2014). The extrinsic cardiac ANS mediates connections between the heart and the nervous system (Figure 4), whereas the intrinsic ANS of the heart consists of a local network of ganglionated plexi, interconnecting ganglia, and autonomic nerve axons (Hou et al., 2007).

Extrinsic sympathetic fibers mainly derive from the sympathetic (i.e., superior and middle cervical, stellate, mediastinal, and thoracic) ganglia (Dacey et al., 2022). The axons of the neurons located herein will give rise to the superior, middle, and inferior cardiac sympathetic nerves, whose terminal branches travel along the coronary vessels from the epicardial regions towards the endocardium of the atria and the ventricles. Extrinsic parasympathetic fibers originate mainly from the nucleus ambiguus of the medulla oblongata and are carried almost entirely within the vagus nerves (Kapa et al., 2010), whose cardiac fibers converge at the “third fat pad”, located between the superior vena cava and the aorta, before arriving to the sinoatrial and atrioventricular nodes (Chiou et al., 1997). Extrinsic cardiac innervation is much more tangled than a simple sympathetic-parasympathetic duet, and there are multiple points within the intrinsic cardiac nervous system where sympathetic and parasympathetic nerves converge (e.g., the vagus nerves also contain sympathetic fibers, and parasympathetic fibers are also found in sympathetic nerves). (McGuirt et al., 1997; Randall et al., 2003; Beaumont et al., 2013; Rajendran et al., 2016; Khan et al., 2019).

The afferent signals involved in cardiac autonomic modulation originate from baro-, chemo-, and multimodal receptors located within the heart and the walls of the great vessels. Afferent neural signals are transmitted from the heart to integration centers located within the intrinsic nervous system, extracardiac intrathoracic ganglia, the spinal cord, and the brain stem, which further regulate the neural output to the heart *via* the sympathetic and parasympathetic nerves. Activation of high-pressure baroreceptors located in the carotid sinus and the aortic arch generate the main input for sympathetic stimulation and parasympathetic withdrawal *via* the arterial baroreceptor reflex, whereas chemoreceptor activation within the carotid and aortic bodies as well as the medulla (by hypoxemia, hypercapnia, various neuropeptides such as bradykinin, substance P, or the calcitonin gene-related peptide) drives sympathetic tone *via* direct signaling to the nucleus of the tractus solitarius and medulla (Floras and Ponikowski, 2015). This also initiates local inflammatory and vascular reactions important in cardiac remodeling (Wang et al., 2014). Low-pressure baroreceptors are mainly located at the veno-atrial convergence and react in response to atrial filling and contraction. When activated, they cause inhibition of the cardiac and peripheral sympathetic ANS (Linz et al., 2019). However, at each level of integration, the system can modulate cardiac activity with numerous efferent feedback loops (Goldberger et al., 2019). In addition, both the heart and the blood vessels are densely innervated by sensory nerve endings that express chemo-, mechano-, and thermos-sensitive receptors. Activation of the receptors by a nociceptive stimulus in settings such as myocardial ischemia has been shown to reduce vagal tone and exhibit ventricular proarrhythmic effects (Premkumar and Raisinghani, 2006; Salavatian et al., 2022). The role of nociceptors activation in atrial arrhythmias is less clear.

The exquisitely complex intrinsic ANS of the heart contains clusters of intrinsic autonomic ganglia that form a complex network of ganglionated plexi, located in the fat pads found at the surface of the atria and the ventricles, interconnecting ganglia, and postganglionic autonomic fibers’ axons (Armour et al., 1997). Despite the tangled nature of the intrinsic ANS of the heart, different ganglionated plexi appear to modulate specific cardiac anatomic regions (Sampaio et al., 2003). Damage to the projections of the parasympathetic sinoatrial node nerves that penetrate the epicardium at the pulmonary vein antrum could explain, for instance, the increased heart rate, that is, recorded following pulmonary vein ablation procedures (Armour et al., 1997). Activation of the neurons located in any of the major ganglionated plexi has been shown to affect not only adjacent tissue, but all cardiac chambers (Yuan et al., 1994). Moreover, a large gradient appears to exist between different atrial regions in the density of cholinergic and adrenergic neurons, forming a highly intricate heterogeneously distributed atrial autonomic neural network (Scridon et al., 2018). In addition, the cardiac ANS displays a cross-linked structure of interconnected ganglionated plexi. These autonomic structures provide fine regional regulation of various cardiac functions (e.g., cardiac automaticity and conductivity) and act as integrating centers that process both centripetal and centrifugal information, coordinate the sympathetic and parasympathetic inputs received from the rest of the cardiac ANS, and modulate the complex interactions between the extrinsic and the intrinsic systems (Hou et al., 2007; Scridon et al., 2018). They are mainly located at the surface of the right (e.g., on the right atrium, at the junction between the inferior vena cava and the right atrium) and the left (at the pulmonary veins ostia) atria, whereas ventricular ganglionated plexi are mainly located at the origins of the aorta and the main branches of the coronary system (Armour et al., 1997). Ganglionated plexi located at the junction between the inferior vena cava and the right atrium have been shown to control the function of the atrioventricular node and inferior atrial tissues (O'Toole et al., 1986; Cardinal et al., 2009).

## Effects of autonomic inputs on cardiac electrophysiology

**FIGURE 5:**
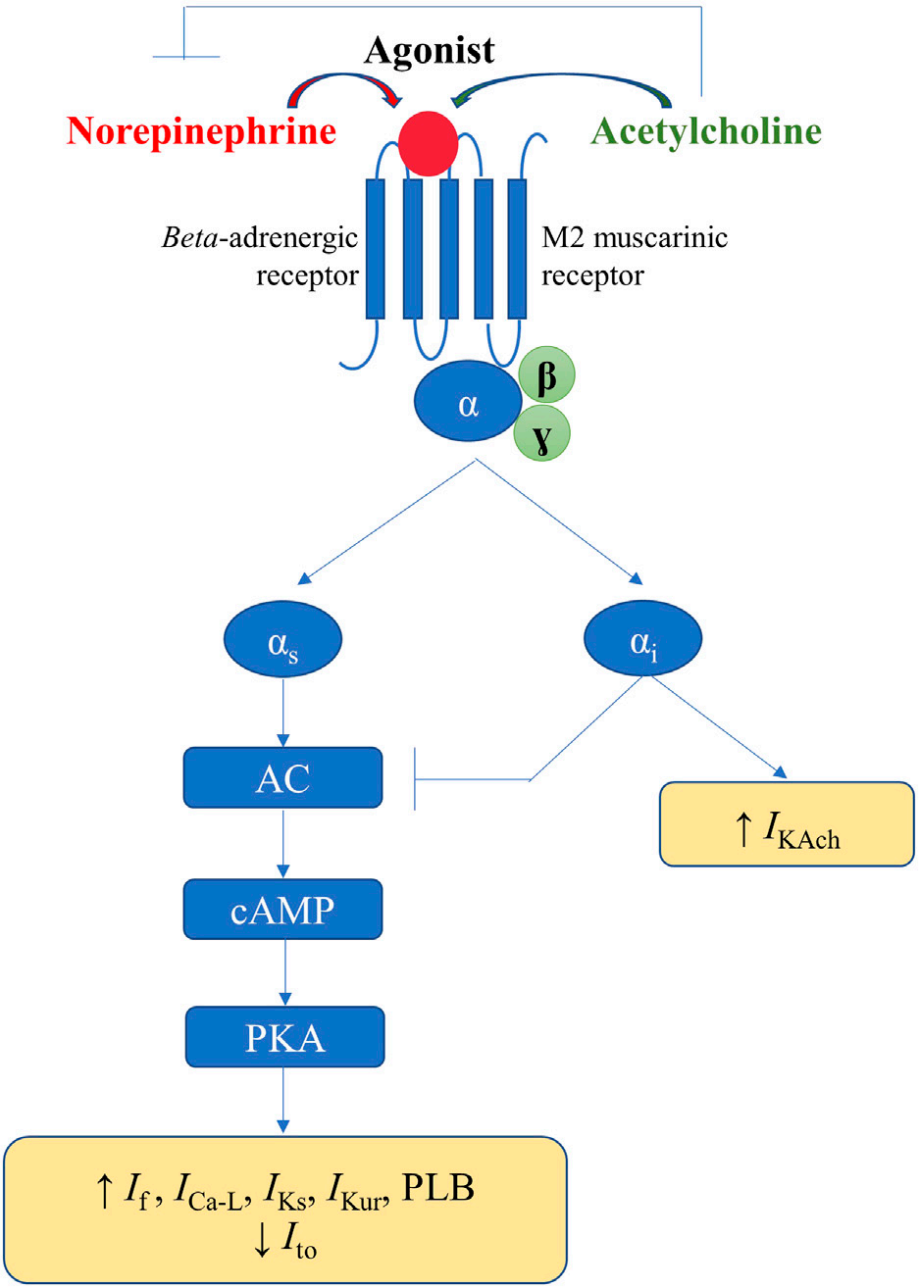
Physiological mechanisms involved in the autonomic modulation of cardiac electrical activity.

In response to the appropriate stimuli, postganglionic sympathetic fibers release norepinephrine, thus activating the cardiac *beta*-adrenergic receptors (particularly *beta*-1), coupled with stimulatory G proteins (Figure 5). Subsequent activation of protein kinase A, *via* the adenylyl cyclase/cAMP signaling pathway, increases the activity of the L-type calcium current (*I*_Ca-L_) and phospholamban, thus increasing calcium inflow, as well as calcium uptake by the SR. Additionally, sympathetic stimulation enhances the activity of the pacemaker current (*I*_f_), inhibits the cardiac transient outward potassium current (*I*_to_), and stimulates the slow delayed rectified potassium current (*I*_Ks_) and the ultra-rapid delayed rectified potassium current (*I*_Kur_), the latter expressed exclusively at the level of the atria (Schotten et al., 2011). Overall, the net result of these effects will be an increase in cardiac chronotropy, excitability, and dromotropy, in parallel with unaffected or only slightly abbreviated action potential, both at the level of the atria and of the ventricles (Figure 5). (Zipes et al., 1974; Vaseghi et al., 2013)

Activation of the parasympathetic nervous system will lead to release of acetylcholine from the nerve endings and to consequent activation of muscarinic (particularly M2) receptors within the heart, triggering effects that counteract those of the cardiac sympathetic nervous system. Alongside the interactions that occur within the intrinsic cardiac nervous system, parasympathetic-sympathetic interferences also occur at other–presynaptic, receptor, and intracellular–levels (Figure 5). Presinaptically, acetylcholine inhibits norepinephrine release. Acetylcholine inhibits the adenylyl cyclase/cAMP system (*via* M2 receptors coupled with inhibitory G proteins), thereby reducing the activity of *I*_Ca-L_ and *I*_f_ (Figure 5). Muscarinic receptors activation and the consequent stimulation of inhibitory G proteins also inhibit, *via* a direct, adenylyl cyclase/cAMP system-independent mechanism, the acetylcholine-gated potassium current (*I*_KAch_), expressed almost exclusively at the level of the atria (Linz et al., 2019). Overall, the net result will be a decrease in cardiac chronotropy, excitability, and dromotropy, in parallel with a significant and heterogeneous shortening in atrial action potential and refractoriness (due to inhomogeneous spatial distribution of atrial M2 receptors and parasympathetic nerve endings) (Figure 5). (Liu and Nattel, 1997; Schotten et al., 2011) Accumulating data also indicate a potential role for neuromediators such as the neuropeptide Y, in control of cardiac function, particularly in progressive cardiac pathology (Armour et al., 2005; Herring et al., 2008; Tan et al., 2018; Ajijola et al., 2020).

Simplistically, sympathetic and parasympathetic nervous systems essentially work in a ‘ying-yang’ fashion, displaying opposite effects on cardiac electrical parameters. However, constant communication exists between the two systems that complicates their effects, and an intricate balance exists between sympathetic and parasympathetic inputs. Greater absolute reductions in heart rate are recorded when parasympathetic stimulation is applied in the presence of higher sympathetic tone, an interaction known as accentuated antagonism (Stramba-Badiale et al., 1991). Similar effects are recorded regarding calcium handling and cardiac electrophysiology parameters (Brack et al., 2004). Meanwhile, both chronic and acute afferent vagus nerve stimulation have been shown to reflexively inhibit efferent sympathetic nerve activity (Schwartz et al., 1973; Shen et al., 2011). Furthermore, in diseased hearts, autonomic stimulation may exert effects opposite to those seen in the normal heart, and vagal (but not sympathetic) stimulation has different effects on atrial and ventricular myocytes (Shen and Zipes, 2014).

## Autonomic imbalance and ectopic atrial activity

### A plethora of intricate mechanisms links atrial arrhythmias risk factors with autonomic imbalance

**FIGURE 6:**
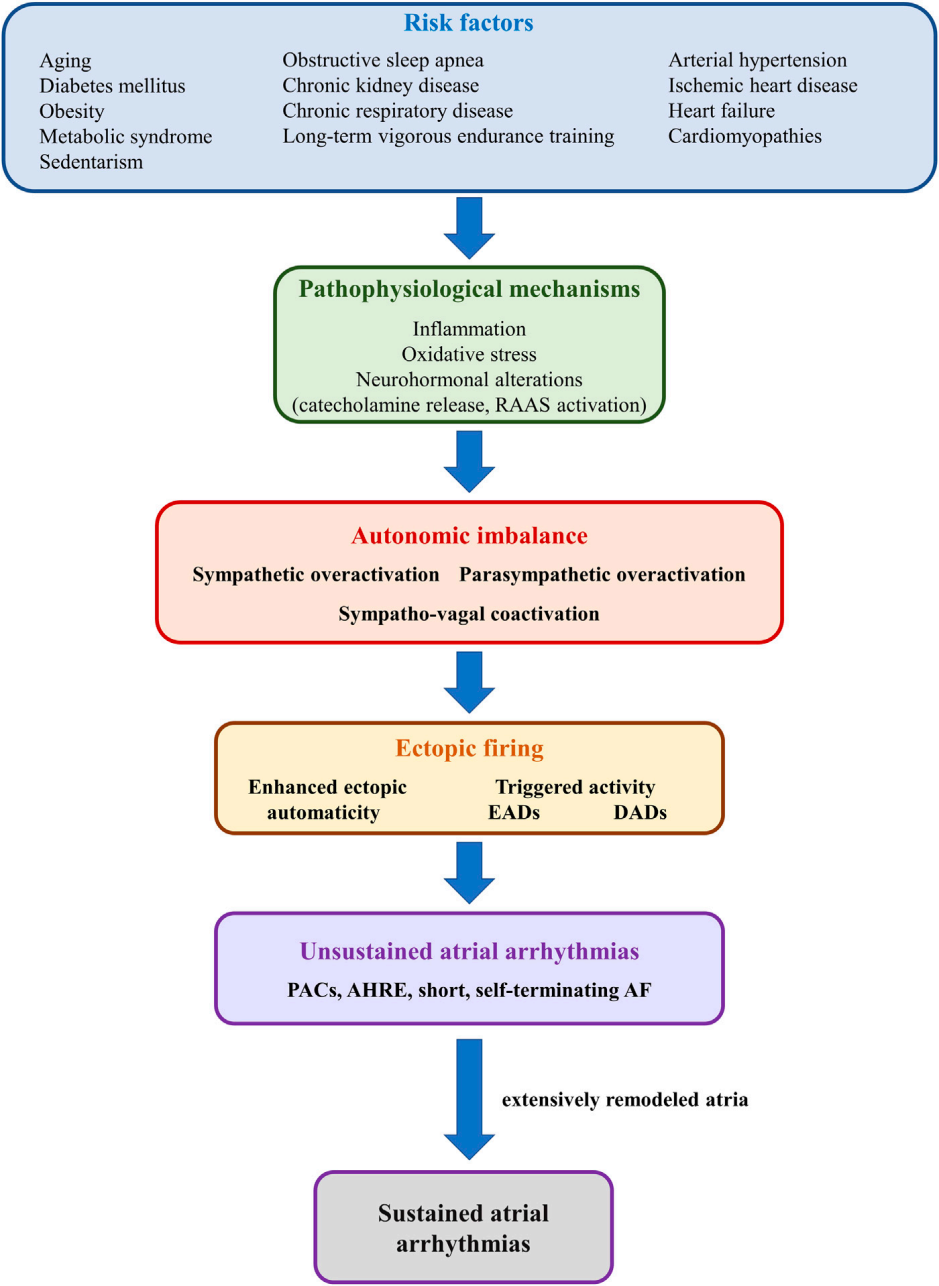
Schematic representation of the mechanisms linking risk factors with autonomic imbalance and atrial ectopic activity. Risk factors such as diabetes mellitus, obesity, or heart failure (*upper panel*) promote, *via* multiple mechanisms (*second panel*), sympathetic-parasympathetic imbalance (*third panel*). In turn, sympathetic or parasympathetic overactivation, or a combination of both, promotes enhanced ectopic automaticity or triggered activity (*fourth panel*), and atrial ectopic activity (*fifth panel*). In the absence of adequate substrate, ectopic activity translates into unsustained atrial arrhythmias, such as premature atrial contractions or short, self-terminating episodes of clinically overt or subclinical atrial fibrillation (*sixth panel*). However, in the presence of extensively remodeled atria, even reduced ectopic activity can initiate sustained episodes of atrial arrhythmia.

A wide array of cardiac and non-cardiac conditions act as major risk factors for atrial arrhythmias, particularly AF, by promoting atrial proarrhythmic electrical and structural remodeling (Şerban and Scridon, 2018; Scridon and Balan, 2022). Numerous systemic (e.g., obesity, diabetes mellitus, hypertension, obstructive sleep apnea, aging, sustained endurance training) and cardiac (e.g., heart failure, cardiomyopathies, acute and chronic ischemic heart disease) conditions have also been shown to induce structural and/or functional autonomic alterations, further promoting atrial arrhythmogenicity (Figure 6). (Scridon et al., 2018) Increased sympathetic tone is a major pathophysiological feature of most of these conditions, although spontaneous unsustained AF has also been associated with sympathetic withdrawal and relative parasympathetic activity in aging, spontaneously hypertensive rats (Scridon et al., 2012). Moreover, in that model, sympathetic stimulation exhibited marked atrial antiarrhythmic effects, whereas parasympathetic stimulation induced a significant increase in atrial arrhythmic burden (Scridon et al., 2012; Sayin et al., 2015). Long-term vigorous endurance training has also been related to vagally-mediated AF (Figure 6). (Elliott et al., 2017) Meanwhile, transient exposure to occasional intensive exercise has been shown to favor atrial arrhythmias *via* a complex sympathetic-parasympathetic interplay. Adaption of the cardiac output to exercise relies on progressive sympathetic activation and concomitant parasympathetic withdrawal. Upon cessation of exercise, sustained sympathetic hyperactivity coupled with rapid parasympathetic reactivation result in a transient, proarrhythmogenic state of sympathetic-parasympathetic coactivation that favors the occurrence of atrial arrhythmias (Elliott et al., 2017). A similar sympatho-vagal coactivation has also been reported in patients with obstructive sleep apnea, in whom increased atrial transmural pressure gradients lead to parasympathetic activation, *via* the diving reflex, whereas pulmonary stretch and hypoxia prompt sympathetic surge (Linz et al., 2018).

The pathophysiological bases of cardiac autonomic neuropathy have not yet been fully elucidated and are likely to vary widely depending on the underlying pathology. Among them, inflammation, oxidative stress, and neurohormonal alterations [particularly catecholamine release and renin-angiotensin-aldosterone system (RAAS) activation], encountered in various degrees in the vast majority of cardiac diseases, appear to play central roles in the development of cardiac autonomic neuropathy and in the pathophysiology of atrial arrhythmias (Figure 6). (Şerban and Scridon, 2018) Additional factors, such as increased production of advanced glycation end-products, hyperglycemic activation of the polyol and protein kinase C pathways, and neurovascular insufficiency, as well as sudden sympathetic activation in response to transient episodes of hypoglycemia may contribute to cardiac autonomic neuropathy and atrial arrhythmia occurrence in the presence of diabetes mellitus (Şerban and Scridon, 2018). Meanwhile, proinflammatory cytokines, released in a wide range of cardiac conditions (Scridon et al., 2015), have been shown to promote restructuring of the cardiac autonomic network, stimulate nerve sprouting and growth, favor autonomic variations, and promote cholinergic transdifferentiation of cardiac sympathetic nerves (Kanazawa et al., 2010). Inflammation is also seen as a link between epicardial adipose tissue (a major local source of cytokines, hormones, and vasoactive substances), autonomic dysfunction, and atrial arrhythmias (Scridon et al., 2015). Proinflammatory adipocytokines have been shown to stimulate ganglionated plexi located within the epicardial fat pads, promoting both sympathetic and parasympathetic overactivation, thus favoring the occurrence of atrial arrhythmias (Scridon et al., 2015). Sympathetic activation has also been shown to induce proinflammatory cytokine expression in the atrial myocardium (Acampa et al., 2018), thus generating a highly proarrhythmic vicious circle at the level of the atria. A similar reciprocal relationship has also been reported between increased adrenergic drive and oxidative stress, as well as between increased adrenergic drive and activation of the RAAS (Acampa et al., 2018).

Additionally, AF itself has been postulated to cause substantial autonomic imbalance, leading to a feedback loop in which autonomic dysfunction contributes to the pathogenesis of AF, whereas AF promotes autonomic dysfunction, further perpetuating and facilitating the progression of the arrhythmia (Figure 6). (Scridon et al., 2018) Long coupling intervals between ventricular contractions during AF have been shown to cause beat-to-beat variations in pulse pressure, causing sympathetic activation and blunting baroreflex sensitivity (Segerson et al., 2007; Malik et al., 2019). In animal models, pacing-induced AF has been shown to cause an increase in both sympathetic and parasympathetic innervation of the atria, while also promoting deafferentation of the atria (Yu et al., 2014; Malik et al., 2019). In addition, during AF, decreased arterial blood pressure and the consequent unloading of arterial baroreceptors, coupled with increased cardiac filling pressures with consequent activation of cardiac baroreceptors “confuse” the ANS, leading to simultaneous, conflicting sympatho-excitatory and sympatho-inhibitory signals (Scridon et al., 2018).

### Role of autonomic dysfunction in the genesis of atrial ectopic activity

Activation of the extrinsic ANS of the heart is seen prior to AF onset in 73%–100% of cases (Tan et al., 2008; Choi et al., 2010), with a predominance of vagal activity in young patients with “idiopathic AF” and structurally normal hearts, and sympathetic dominance in patients with “organic AF” or following cardiac surgery (Zhong et al., 2022). Diurnal prevalence, increased sympathetic indices at heart rate variability (HRV) analysis, and the presence of frequent PACs that produce a short-long-short RR intervals pattern provide indirect evidence for a strong adrenergic stimulation-atrial ectopy-AF relationship (Lombardi et al., 2004). Oppositely, nocturnal prevalence and increased parasympathetic indices at HRV analysis underscore the implication of the vagal system in arrhythmia onset (Herweg et al., 1998). Meanwhile, a direct implication of the intrinsic cardiac ANS in atrial arrhythmias occurrence is much less clear. Studies performed in dogs using direct nerve activity recordings from both intrinsic and extrinsic autonomic components showed that although the vast majority of AF episodes were preceded by simultaneous firings from both the intrinsic and extrinsic autonomic systems, ≈10% of the episodes were triggered by activation of the intrinsic cardiac ANS alone (Yu et al., 2014). An increase in PAC burden has been noted following increased sympathetic activity (Honjo et al., 2003), whereas the contribution of autonomic dysfunction to atrial high rate episodes (AHRE) is much less clear. The sole study performed to date showed no correlation between HRV indices and the burden of AHRE (Khan et al., 2021). However, that study was small, with only 22 patients included in each group, and there was no control group. Models of neurally-induced arrhythmias have also been developed and have been very useful in defining effects of surgical, pharmacological, and bioelectric interventions (Armour et al., 1972; Cardinal et al., 2006; Richer et al., 2008; Gibbons et al., 2012; Salavatian et al., 2016).

Vagal activation exerts different electrophysiological effects at the level of the atria and the ventricles. At ventricular level, vagal stimulation prolongs action potential duration and refractoriness, exhibiting antiarrhythmic effects. Meanwhile, at the atrial level, vagal stimulation causes opposite effects, abbreviating atrial action potentials and refractoriness in a spatially heterogeneous fashion, thus setting the background (dispersion of refractoriness) for reentry and atrial arrhythmias (Shen and Zipes, 2014). In addition, vagally-released non-cholinergic molecules such as the vasoactive intestinal peptide further shorten the atrial action potential and generate intraatrial conduction delays. Vagal stimulation has been shown to promote not only reentry, but also atrial ectopic firing, by promoting early afterdepolarization toward the end of phase 3 of the action potential and allowing ectopic automaticity to arise (Figure 6). (Burashnikov and Antzelevitch, 2003) In an early study by Scherf et al. (1950) local acetylcholine application was followed by rapid action potentials firing and AF, whereas arrhythmias offset was recorded when the parasympathetic source was removed. In dogs, high-frequency stimulation of the cervical vagal trunk and of the atrial epicardial ganglionated plexi also significantly increased AF occurrence, effect that could be eliminated by blocking vagus nerves function with atropine (Lu et al., 2009; Qin et al., 2019). The exact mechanisms by which parasympathetic activation triggers atrial ectopic activity remain to date unclear. However, by decreasing *I*_f_ activity in the sinus node, vagal stimulation can alter the normal pacemaking hierarchy of the heart and can thus enable ectopic activity to arise (Scridon et al., 2018). In addition, by activating *I*_KAch_, vagal stimulation can also shorten the action potential and thus promote the occurrence of late-phase 3 afterdepolarizations (Figure 6), particularly if accompanied by an increase in the calcium transient (Burashnikov and Antzelevitch, 2003). The impact of vagus nerve stimulation on atrial arrhythmias is highly dependent on stimulation parameters. Both vagus nerve and spinal cord stimulation have been shown to exhibit stabilizing effects on intrinsic cardiac nervous system function, which affects ANS contribution to arrhythmia formation (Cardinal et al., 2006; Gibbons et al., 2012; Salavatian et al., 2016).

Sympathetic activation is recognized as the most relevant culprit in autonomic dysfunction-related atrial ectopy (Coumel, 1994), and has been associated with increased risk of PACs, AF, and, more recently, with the presence of AHRE (Yılmaz, 2021). Whereas stimulation of cardiac *beta*-adrenoreceptors augments *I*_f_, *alpha*-receptor stimulation has been shown to decrease *I*_K1_ activity. Together, these responses can augment automaticity in non-pacemaker atrial cells, leading to atrial ectopic activity, as demonstrated in rat pulmonary veins (Figure 6). (Maupoil et al. 2007) In parallel, by increasing the activity of *I*_Ca-L_ as well as that of phospholamban, sympathetic stimulation increases both calcium inflow through sarcolemmal voltage‐gated calcium channels and calcium uptake by the SR, thereby promoting SR calcium overload. These changes, accompanied by simultaneous sympathetic-induced increase in RyR2 activity, leads to excessive intracellular calcium accumulation, with consequent activation of the sarcolemmal NCX. Due to its 3:1 stoichiometry, with inflow of three sodium ions for each ion of calcium expelled from the cell, NCX functioning is electrogenic, generating a net inward cation current that can underlie delayed afterdepolarizations-related ectopic activity (Linz et al., 2019). *Alpha*-adrenergic-induced inhibition of *I*_K1_ coupled with sympathetic-induced cardiomyocyte calcium overload thereby ensure both action potential prolongation and a net gain of cations, favoring the occurrence of early afterdepolarizations in atrial cells (Scridon et al., 2018).

Complex interactions exist between the two ANS limbs, and atrial ectopic firing is rarely purely sympathetic or vagally mediated. Studies have shown that sympathetic and parasympathetic coactivation often precedes atrial ectopic activity, in both clinical and experimental settings. An increase in cardiac sympathetic modulation minutes before, followed by parasympathetic activation immediately prior to arrhythmia onset have been reported in paroxysmal AF patients (Bettoni and Zimmermann, 2002). Simultaneous sympathetic and parasympathetic discharges have also been shown to precede paroxysmal AF onset in dog models of rapid atrial pacing (Tan et al., 2008) and heart failure (Ogawa et al., 2007), whereas bilateral cryoablation of the stellate ganglia and of the superior cardiac branches of the left vagus nerve eliminated all AF episodes (Bettoni and Zimmermann, 2002; Ogawa et al., 2007). Similarly, AF onset was preceded by synchronous sympathetic-parasympathetic activation in the study by Tan et al. (2008) Moreover, AF inducibility was significantly higher in that study when a combination of isoprenaline and acetylcholine was administered compared to acetylcholine alone (Tan et al., 2008), suggesting that sympathetic-vagal coactivation is more arrhythmogenic than vagal activation alone. The pathogenic pathway connecting sympatho-vagal coactivation with atrial ectopy is rather intuitive. Increased calcium transient caused by sympathetic activation accompanied by vagal-induced atrial effective refractory period shortening disrupts the balance between action potential duration and intracellular calcium transient, cardiac electrical features that are normally closely coupled (Amar et al., 2003). This further leads to increased NCX activity, thus favoring early afterdepolarizations and atrial triggered activity.

## Gaps in evidence and future research

The clinical impact of AF is well known (Hindricks et al., 2021). Meanwhile, that of PACs, and particularly of AHRE, remains elusive. Electroanatomical mapping of AF recurrence following spontaneous or electrical restoration of sinus rhythm indicates PACs as the most common trigger of recurrent AF episodes (Haïssaguerre et al., 2000). Frequent PACs and runs of non-sustained atrial tachyarrhythmias have also been associated with increased risk of incident AF (Dewland et al., 2013; Nguyen et al., 2016; Christensen et al., 2017; Kerola et al., 2019) and of AF-related complications such as stroke and heart failure (O'Neal et al., 2017). In a rat model of spontaneous AF, short, non-sustained runs of AF have been associated with increased endocardial von Willebrand factor expression and intraatrial thrombosis (Scridon et al., 2013). Very short (i.e., ≤10–20 s/day) episodes of AHRE do not appear to be clinically relevant. Longer episodes (i.e., ≥5–6 min) have been associated with higher risk of clinically overt AF, stroke or systemic embolism, and cardiovascular events, including death. A linear relationship has been described between AHRE duration and risk of stroke (Hindricks et al., 2021). Whether AHRE are only a risk marker (not causally associated) or a risk factor of stroke *per se* remains a matter of debate. The potential clinical benefit of oral anticoagulation in these patients also remains unclear.

The role of autonomic imbalance in atrial ectopy occurrence has been well established over the years. Moreover, autonomic imbalance has been related to the presence and severity of symptoms (i.e., dizziness, presyncope, and syncope) in these patients (Linz et al., 2019). Pharmacologic or interventional ANS modulation could thus emerge as a viable target for maintenance of sinus rhythm as well as for symptoms control. Numerous questions still remain to be answered regarding the autonomic imbalance-atrial ectopy relationship.

Data regarding autonomic imbalance in animal models and patients with atrial ectopy indicate high inter- and intra-individual variability in the magnitude, the contribution, and even the direction of autonomic imbalance in this setting. Observational clinical studies yielded conflicting results, likely due to the inclusion of heterogeneous and insufficiently well characterized populations, usage of different assessment techniques, analysis of different parameters, and different criteria used to diagnose autonomic imbalance and atrial ectopic activity. Furthermore, although observational studies can provide invaluable insights into the ANS-atrial arrhythmias relationship, they cannot ascribe causality.

Animal studies can generally provide much more robust mechanistic insights. More complex and more accurate methods can be used in preclinical studies and, unlike in clinical studies, selective and specific sympathetic and parasympathetic manipulation can be applied in experimental settings (Scridon et al., 2012; Sayin et al., 2015; Scridon et al., 2021). However, caution should be used when extrapolating animal data to human patients. Interspecies differences should obviously be considered. More importantly, currently available experimental atrial arrhythmia models may not be the most appropriate for ANS assessment. Whereas most atrial arrhythmia patients are of advanced age, have coexisting conditions, such as hypertension, heart failure, obesity, or ischemic heart disease, and are often on different cardioactive medications, experimental studies most commonly use juvenile, healthy, and medication-free animals. Such differences raise questions regarding the translational value of these preclinical models. Moreover, rapid atrial pacing is generally employed for mimicking atrial arrhythmias in experimental settings. Although highly efficient, pacing the atria at 1,000 bpm or more will inevitably produce autonomic changes, hindering our ability to accurately quantify the intensity of autonomic activation, the sympathetic-parasympathetic interactions, and their role in the occurrence of atrial arrhythmias. Future experimental studies will therefore have to employ more clinically relevant atrial arrhythmia models in order to allow better translation of the results to the clinical setting.

Contrary to the experimental settings, accurate evaluation of the ANS is highly challenging in clinical settings. Baroreflex sensitivity, heart rate recovery, heart rate responses to physiological manipulations (i.e., Ewing’s tests), and particularly HRV analysis using continuous ECG recordings are techniques that are non-invasive and relatively easy to use (Table 1). However, the results may be affected by the presence of an implanted pacemaker, of AF, or other cardiac arrhythmias, and they provide only indirect information regarding ANS activity. One common feature of all these methods is that they all imply evaluation of ANS modulation of the sinus node, whereas the adverse effects of autonomic imbalance in atrial arrhythmias are mainly driven by its direct impact at the level of the atria. In addition, the results are affected by the presence of concomitant sinus node disease, which is rather common with advancing age or in patients with obesity, hypertension, obstructive sleep apnea, or heart failure (Piccirillo et al., 2009; Floras and Ponikowski, 2015; Linz et al., 2019). Moreover, the complex anatomic/physiological relationships within the cardiac ANS, the complex interplay within this multilevel system (including multiple levels of feedback and excitatory/inhibitory control), regional autonomic effects that likely exist in the presence of cardiac autonomic neuropathy as well as possibly present in healthy subjects, raise questions about the ability of these markers to accurately quantify cardiac sympathetic/parasympathetic modulation or guide therapeutic management. These challenges explain why none of these techniques has entered the realm of clinical evaluation even after many decades of evaluation.

**TABLE 1:** Tests of cardiac autonomic function with potential usefulness in patients with atrial arrhythmias.

Test	Physiological meaning
Heart rate	Autonomic impact on the sinus node
Heart rate variability	Autonomic modulation of the sinus node
Heart rate recovery	Parasympathetic reactivation following cessation of physical exercise
Baroreflex sensitivity	Response of the sinus node to baroreceptor activation
Autonomic reflex testing (Ewing’s tests)	Heart rate responses to physiological manipulations (i.e., breathing, handgrip, tilting, Valsalva maneuvers)
Plasma/urinary catecholamines levels	Total catecholamine spillover to plasma or urine
Transcardiac norepinephrine spillover	Cardiac spillover of norepinephrine
Sympathetic nerve recordings by microneurography	Evaluation of regional (muscle or skin) sympathetic output
PET/CT evaluation of cardiac autonomic nerves	Distribution and function of cardiac sympathetic nerves

**TABLE 2:** Cardiac neuromodulation interventions with potential applications in patients with atrial arrhythmias.

Pharmacological and biological interventions	Interventional neuromodulation techniques	
	Ablative/inhibition-based strategies	Stimulation-based strategies
*Beta*-adrenoreceptor blockade (metoprolol)	Surgical ablation of GP	Spinal cord stimulation
Central sympathetic inhibition (moxonidine)	Transcatheter endocardial and/or epicardial ablation of GP (with or without concomitant PVI)	Low-level cervical vagus nerve stimulation
RAAS blockade	Selective atrial vagal denervation	Low-level transcutaneous tragus stimulation
Colchicine	Ablation of extrinsic cardiac nerves	Carotid baroreceptor stimulation
Statins	Transcutaneous blockade of the stellate ganglia	—
Long-chain n-3 polyunsaturated fatty acids	Catheter-based renal sympathetic denervation	—
Probucol	—	—
Botulinum toxin	—	—
G-protein inhibitory peptides	—	—
Gene therapy (G*α*i2 C- and G*α*01 C-terminal peptide delivery)	—	—

GP, ganglionated plexi; PVI, pulmonary vein isolation; RAAS, renin-angiotensin-aldosterone system.

A plethora of other ANS evaluation methods have been developped over the years (Table 1), including measurement of circulating and urinary catecholamines levels, direct muscular or skin sympathetic nerve recordings by microneurography, measurement of transcardiac norepinephrine spillover, and PET/CT evaluation of autonomic nerves activity. Although such methods could provide a more precise estimation of ANS activity, these techniques are complex and difficult to use in routine clinical practice, require dedicated costly equipment, and are unavailable in most centers. Moreover, the clinical value of routine assessment using these methods in improving patient phenotyping and therapeutic management remains unclear. None are capable of assessing the ANS in its entirety. Future studies need to establish the potential value of these techniques and/or identify other non-invasive, easy to use markers to evaluate cardiac autonomic modulation, similarly to what has been achieved in other clinical settings (Scridon and Şerban, 2016; Delinière et al., 2019).

Finally, studies will have to establish the clinical impact of pharmacologic and interventional neuromodulation for rebalancing cardiac autonomic function in patients with atrial ectopy (Table 2). Modification of risk factors known to be associated with cardiac autonomic neuropathy, such as obesity, sedentarism, hypertension, diabetes mellitus, or obstructive sleep apnea has been shown to efficiently reduce AF burden, particularly when a global approach of risk factor modification is employed (Lau et al., 2017).

*Beta*-adrenoreceptor blockade using metoprolol, central sympathetic inhibition using moxonidine, RAAS blockade, colchicine, statins, long-chain n-3 polyunsaturated fatty acids, and probucol have all been shown to modulate sympathetic activity and to decrease atrial arrhythmogenicity in some clinical and preclinical settings (Kühlkamp et al., 2000; Naccarelli et al., 2007; Fauchier et al., 2008; Gong et al., 2009; Van Wagoner, 2011; Giannopoulos et al., 2014; Kerola et al., 2019), although none of them has, to date, demonstrated sufficient efficacy to become an established part of clinical practice for atrial arrhythmias prevention and/or treatment. More novel pharmacological and biological methods such as injection of botulinum toxin, which interferes with cholinergic neurotransmission, in the ganglionated plexi at the time of open-heart surgery and selective disruption of parasympathetic signaling using G-protein inhibitory peptides, have also shown promising effects in preclinical studies (Aistrup et al., 2009; Pokushalov et al., 2015), but confirmation in clinical settings is required. Alternative techniques, such as acupuncture, have also been tested, with some data indicating a potential benefit (Lombardi et al., 2012). Delivery to the posterior left atrium of plasmids containing cDNA for the G*α*i2 C-terminal peptide, particularly when coupled with G*α*01 C-terminal peptide delivery, led to massive, atrial-selective attenuation of vagal signaling, opening the way for genetic therapy in atrial arrhythmias and neuropathy (Bauer et al., 2004).

Surgical and transcatheter ablation of endocardial and/or epicardial ganglionated plexi, selective atrial vagal denervation, extrinsic cardiac nerves ablation, transcutaneous stellate ganglia blockade with lidocaine, spinal cord stimulation, renal sympathetic denervation, low-level cervical vagus nerve or tragus transcutaneous stimulation, and carotid baroreceptor stimulation using implantable devices have reduced AF burden in clinical and preclinical studies. Nevertheless, many results remain scarce or highly controversial, and long-term outcomes remain questionable (Lemery et al., 2006; Ogawa et al., 2009; Sakamoto et al., 2010; Bernstein et al., 2012; Zhao et al., 2012; Katritsis et al., 2013; Driessen et al., 2016; Goldberger et al., 2019). The long-term efficacy and safety, the optimal ablation/stimulation protocols, as well as the criteria for selecting the most adequate target population, for establishing the optimal time to intervene, and for confirming successful autonomic modification also remain to be defined. In addition, the long-term impact of these approaches on ‘hard’ clinical endpoints (e.g., stroke, heart failure, or mortality) remains unknown. Moreover, the impact of such approaches has never been tested in patients with frequent PACs or AHRE. If efficient, these techniques may reduce AF prevalence and, consequently, the burden that AF and AF-related complications impose on the healthcare systems. Adequately powered, well-conducted randomized controlled trials are needed to clarify all these issues.

## Conclusion

The heart is one of the most richly innervated organs and the extrinsic and intrinsic cardiac ANS provides fine tuning of cardiac electrophysiology. Atrial ectopic activity can arise *via* multiple mechanisms promoted by sympathetic or vagal fluxes, or by a combination of both. Furthermore, a bidirectional relationship contributes to the pathogenesis of atrial arrhythmias, whereby autonomic imbalance promotes atrial arrhythmic events, which, in turn, promote atrial autonomic imbalance. Autonomic modulation has thus become a major target of investigation over the past decades. Combining aggressive risk factors management together with adjunct pharmacological or interventional strategies may provide a solution for rebalancing autonomic tone and for reducing atrial arrhythmic burden. Numerous questions need to be answered before reaching this milestone. Techniques that are accurate, widely available, non-invasive, and easy-to-use remain to be developed and implemented in routine clinical practice. With very few exceptions, data regarding pharmacological and interventional neuromodulation strategies are limited, controversial, and mostly derived from small-scale studies. The cardiac ANS is unique in each individual and the autonomic imbalance-atrial arrhythmia relationship is highly complex. A personalized approach, using patient-specific, targeted correction of autonomic abnormalities may thus be needed to reduce the atrial arrhythmia burden. Future studies from basic and clinical laboratories will have to clarify the exact role of autonomic imbalance in atrial arrhythmogenesis, to elucidate whether interventions targeting specific components of cardiac autonomic innervation can improve atrial arrhythmias management, and to identify the optimal time and means for intervention.

## Abbreviations

AF, atrial fibrillation; AHRE, atrial high rate episodes; ANS, autonomic nervous system; HRV, heart rate variability; *I*_Ca-L_, L-type calcium current; *I*_f_, pacemaker current; *I*_KAch_, acetylcholine-gated potassium current; *I*_Ks_, slow delayed rectified potassium current; *I*_Kur_, ultra-rapid delayed rectified potassium current; *I*_to_, transient outward potassium current; NCX, Na^+^/Ca^2+^ exchanger; PAC, premature atrial contraction; RyR2, ryanodine-receptor channels; SR, sarcoplasmic reticulum.
